# Molecular Identification of Shark Meat From Local Markets in Southern Brazil Based on DNA Barcoding: Evidence for Mislabeling and Trade of Endangered Species

**DOI:** 10.3389/fgene.2018.00138

**Published:** 2018-04-27

**Authors:** Fernanda Almerón-Souza, Christian Sperb, Carolina L. Castilho, Pedro I. C. C. Figueiredo, Leonardo T. Gonçalves, Rodrigo Machado, Larissa R. Oliveira, Victor H. Valiati, Nelson J. R. Fagundes

**Affiliations:** ^1^Laboratório de Genética Médica e Evolução, Departamento de Genética, Universidade Federal do Rio Grande do Sul, Porto Alegre, Brazil; ^2^Laboratório de Biologia Molecular, Centro de Ciências da Saúde, Universidade do Vale do Rio dos Sinos, São Leopoldo, Brazil; ^3^Laboratório de Ecologia de Mamíferos, Centro de Ciências da Saúde, Universidade do Vale do Rio dos Sinos, São Leopoldo, Brazil

**Keywords:** cação, elasmobranch, cytochrome oxidase-1, shark fisheries, wildlife DNA forensics

## Abstract

Elasmobranchs, the group of cartilaginous fishes that include sharks and rays, are especially vulnerable to overfishing due to low fecundity and late sexual maturation. A significant number of elasmobranch species are currently overexploited or threatened by fisheries activities. Additionally, several recent reports have indicated that there has been a reduction in regional elasmobranch population sizes. Brazil is an important player in elasmobranch fisheries and one of the largest importers of shark meat. However, carcasses entering the shark meat market have usually had their fins and head removed, which poses a challenge to reliable species identification based on the morphology of captured individuals. This is further complicated by the fact that the internal Brazilian market trades several different elasmobranch species under a common popular name: “cação.” The use of such imprecise nomenclature, even among governmental agencies, is problematic for both controlling the negative effects of shark consumption and informing the consumer about the origins of the product. In this study, we used DNA barcoding (mtDNA, COI gene) to identify, at the species level, “cação” samples available in local markets from Southern Brazil. We collected 63 samples traded as “cação,” which we found to correspond to 20 different species. These included two teleost species: *Xiphias gladius* (*n* = 1) and *Genidens barbus* (*n* = 6), and 18 species from seven elasmobranch orders (Carcharhiniformes, *n* = 42; Squaliformes, *n* = 3; Squatiniformes, *n* = 2; Rhinopristiformes, *n* = 4; Myliobatiformes, *n* = 3; Rajiformes, *n* = 1; and Torpediniformes, *n* = 1). The most common species in our sample were *Prionace glauca* (*n* = 15) and *Sphyrna lewini* (*n* = 14), while all other species were represented by four samples or less. Considering IUCN criteria, 47% of the elasmobranch species found are threatened at the global level, while 53% are threatened and 47% are critically endangered in Brazil. These results underline that labeling the meat of any shark species as “cação” is problematic for monitoring catch allocations from the fishing industry and discourages consumer engagement in conservationist practices through informed decision-making.

## Introduction

Elasmobranch (subclass Elasmobranchii) is a group of cartilaginous fishes that include sharks (superorder Selachii) and rays (superorder Batoidea). Even though elasmobranchs comprise less than 1% of the world fisheries catch (Food and Agriculture Organization of United Nations, 2014, 2016), these species have biological characteristics that make them particularly vulnerable to overfishing, such as a low fecundity and late sexual maturation (Bornatowski et al., 2014b). Indeed, several recent reports have indicated that there has been a reduction of elasmobranch populations, resulting in demographic collapse at a regional scale (Baum et al., 2003; Barausse et al., 2014). The overfishing of sharks is especially problematic because these top predators play a key role in marine ecosystems, and, therefore, their population dynamics may affect all local marine diversity (van der Elst, 1979; Heithaus et al., 2008; Gallagher et al., 2012; Pauly et al., 2013; Worm et al., 2013; Bornatowski et al., 2014a). In 1999, FAO (Food and Agriculture Organization) launched an international plan for the conservation and management of sharks and rays, recognizing the high vulnerability of these organisms (Vannuccini, 1999). However, despite this initiative, a significant number of elasmobranch species has remained overexploited or threatened by fisheries activities (Camhi et al., 2009; Cosandey-Godin and Morgan, 2011), which is illustrated by the 42% global increase in the shark meat trade from 2000 to 2011 (Food and Agriculture Organization of United Nations, 2015).

While shark fins are considered to be one of the most valuable products in the ocean (Gallagher and Hammerschlag, 2011), shark meat often attains only 20–60% of the price of tuna and mackerel meat (Bonfil, 1994). As a result, captured individuals usually have their fins removed for the shark fin market, the head is discarded, and the remaining central body part (“cigar”) is then sold for the shark meat market with no special care (Kotas et al., 2008; Ward-Paige et al., 2012). From a taxonomic point of view, the removal of the head and fins represents a challenge to reliable species identification based on morphological features, allowing shark carcasses to be traded fraudulently (Holmes et al., 2009).

Brazil is among the six countries that have the highest capture rate for elasmobranchs (Lack and Sant, 2006), even though a thorough assessment of the impact of industrial fishing is made difficult by inaccurate records (Barreto et al., 2017). Southern Brazil is a region of high elasmobranch diversity (Lucifora et al., 2011), and has a large extractive marine fishing industry, with approximately 160 thousand metric tons of fish caught annually (MPA. Boletim estatístico da pesca e aquicultura, 2011). The two southernmost states, Santa Catarina (SC) and Rio Grande do Sul (RS), are responsible for 98% of the catches (MPA. Boletim estatístico da pesca e aquicultura, 2011). In addition, Brazil is a major player in the meat trade market, acting as the world's largest importer of shark meat in 2011 (Food and Agriculture Organization of United Nations, 2015). Internally, the Brazilian shark meat market trades several different elasmobranch species under the popular name “cação” (or other related popular terms such as “caçonete” and “anjo”), which is used to label several species (Figure S1). For example, Neto (2013) found 21 different species traded under the common name “cação”, including hammerhead sharks (*Sphyrna* spp.), the blue shark (*Prionace glauca*), the tiger shark (*Galeocerdo cuvier*), the bull shark (*Carcharhinus leucas*), the Galapagos shark (*C. galapagensis*), and the blacktip shark (*C. limbatus*). Consumers value “cação” meat for its low cost and for being a “thornless fish” (Bornatowski et al., 2007). However, most consumers are not aware that “cação” is a synonym for sharks (or rays), and others believe that “cação” represents “a specific race of sharks” or even “a race of small sharks” (Bornatowski et al., 2015). Supermarkets, fisheries, and restaurants often omit any other information when selling “cação” meat. Indeed, the use of this term is so widespread that even Brazilian regulatory agencies categorize all elasmobranch species as “cação” without any species-specific information (MPA. Boletim estatístico da pesca e aquicultura, 2011).

The imprecise nomenclature of elasmobranchs makes it difficult to mitigate the negative effects of human shark consumption, as it becomes more difficult to inform the consumer if the product comes from a threatened species or from an illegal species trade. Since shark carcasses are sliced before being sold, it is virtually impossible to obtain accurate species diagnosis based on morphological traits for marketed elasmobranchs (Bornatowski et al., 2015). Therefore, there is an increasing need for fast, reliable, and cheap testing for determining the taxonomic identity of commercialized fishes (Rasmussen and Morrissey, 2008). A precise identification of marketed species also assures that the correct information is presented to the consumer, motivating him or her to take part in honest and regulated trade (Moretti et al., 2003; Martinez et al., 2005).

DNA barcoding uses a small fragment from a DNA sequence located within a standardized region of the genome to allow precise species identification (Hebert et al., 2003). In animals, the standard DNA barcode comes from a stretch of 650 base pairs (bp) from the 5′ end of the mitochondrial gene Cytochrome Oxidase Subunit I (COI or Cox 1) (Meyer and Paulay, 2005; Hajibabaei et al., 2007). This technique has been widely used in a range of studies of species identification (e.g., Meyer and Paulay, 2005; Lowenstein et al., 2010; Carvalho et al., 2011; Rodrigues-Filho et al., 2012; Galimberti et al., 2013). Whilst DNA barcoding is a valuable tool for species identification, especially when the entire organism cannot be accessed for morphology, there are important limitations concerning its accuracy, which depend on the reference database available and on the degree of genetic difference among species (see Frézal and Leblois, 2008 for a review on the pros and cons of DNA barcoding). The aim of this study is to use DNA barcodes to identify, at the species level, samples of “cação” (or similarly labeled) meat available in local markets in Southern Brazil. Finally, we discuss the implications of these findings in the context of elasmobranch conservation in Brazil.

## Materials and methods

### Sample collection

**Figure 1:**
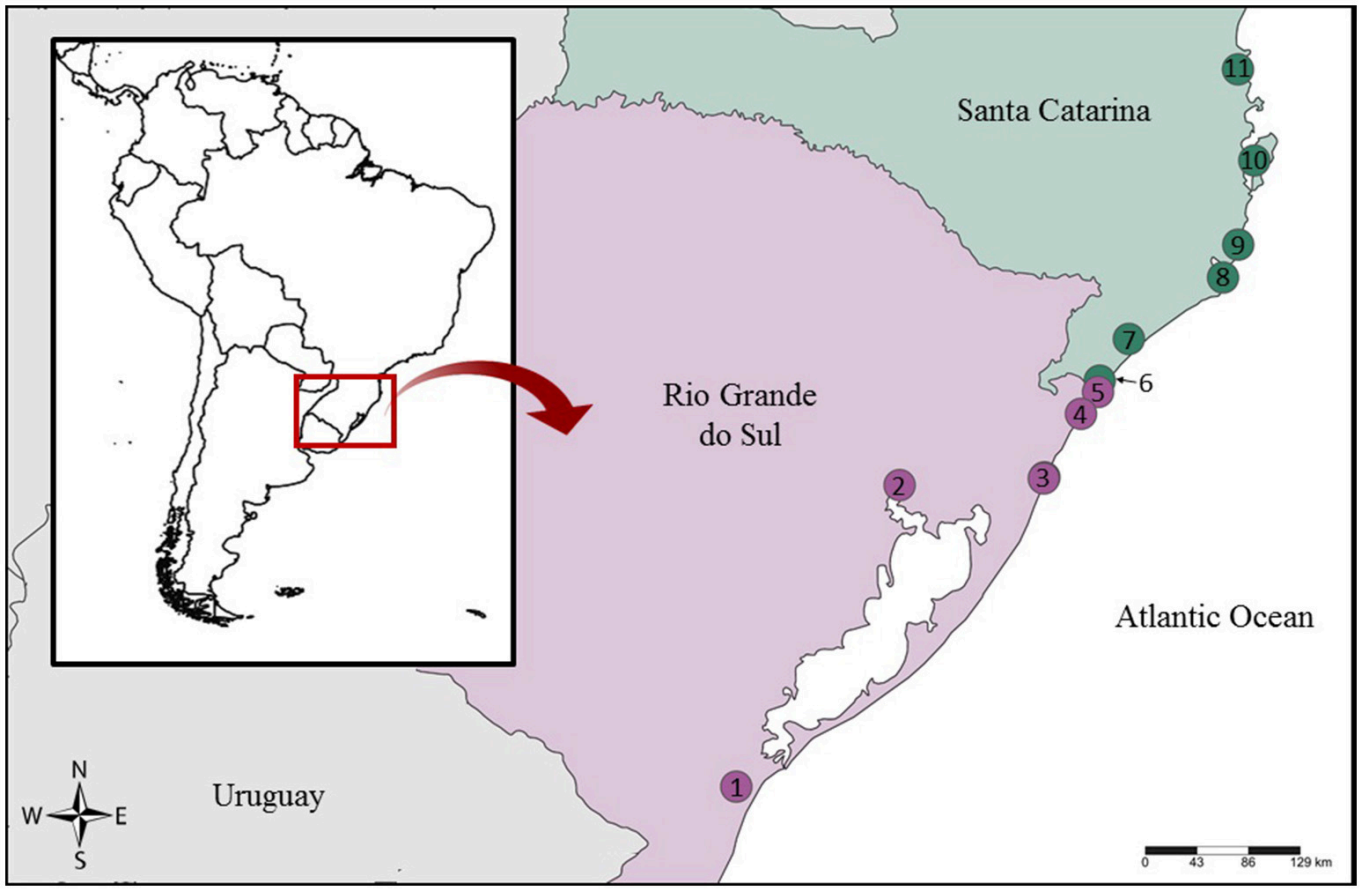
Sampling locations in Southern Brazil. 1, Rio Grande; 2, Porto Alegre; 3, Tramandaí + Imbé; 4, Arroio do Sal; 5, Torres; 6, Passo de Torres; 7, Araranguá; 8, Laguna; 9, Imbituba; 10, Florianópolis; 11, Itajaí.

We studied samples sold under general names such as “cação,” “caçonete,” and “filé anjo,” which usually refer to elasmobranch species. Between 2008-2013 and in 2016 we acquired filet samples from local fish markets and supermarkets in different cities from the RS and SC states in Southern Brazil (Figure 1,Table 1). We also included in the analysis samples from *Sphyrna lewini* (*n* = 4), *Pseudobatos horkelii* (*n* = 2), *Rhizoprionodon lalandii* (*n* = 1), *Narcine brasiliensis* (*n* = 1), *Zapteryx brevirostris* (*n* = 2), and *Gymnura altavela* (*n* = 1), collected from fishing vessels and morphologically identified according to Figueiredo (1977), to serve as controls for the DNA barcode identification. These samples are identified as E__ in Table 1. All samples were stored in 95% ethanol at −20°C.

**Table 1:** Sample information, species identification, average genetic distance, and results from the BLAST search.

**Sample**					**Candidate species**	**Avg. distance^a^**	**% Coverage^b^**	**% Identity^b^**
**ID**	**Accession no**.	**Seq. Size**	**Location**	**Type**				
IIL04	MG703512	650b	Itajaí, SC	fresh	*Carcharhinus brachyurus*	0.001	98	99
IIL05	MG703513	650b	Itajaí, SC	fresh	*Carcharhinus brachyurus*	0.001	97	99
IIL14	MG703514	650b	Itajaí, SC	fresh	*Carcharhinus brachyurus*	0.001	98	99
IIL04-2	MG703515	642b	Itajaí, SC	fresh	*Carcharhinus falciformis*	0.003	98	99
IIL27	MG703516	650b	Itajaí, SC	fresh	*Carcharhinus falciformis*	0.003	99	99
FA08	MG703517	650b	Porto Alegre, RS	fresh	*Galeorhinus galeus*	0.001	95	99
MP60	MG703518	650b	Porto Alegre, RS	fresh	*Genidens barbus*	NC	96	100
E14^*^	MG703519	615b	Arroio do Sal, RS	fresh	*Gymnura altavela*	0.020	99	99
IIL37	MG703520	650b	Laguna, SC	fresh	*Gymnura altavela*	0.021	98	99
IIL36	MG703521	650b	Laguna, SC	fresh	*Myliobatis goodei*	0.013	96	99
E13^*^	MG703522	650b	Torres, RS	fresh	*Narcine brasiliensis*	0.003	99	99
IIL15	MG703523	650b	Itajaí, SC	fresh	*Prionace glauca*	0.001	99	99
IIL30	MG703524	650b	Imbituba, SC	fresh	*Prionace glauca*	0.000	99	99
IIL31	MG703525	613b	Imbituba, SC	fresh	*Prionace glauca*	0.000	100	100
IIL34	MG703526	650b	Imbituba, SC	fresh	*Prionace glauca*	0.000	100	99
IIL35	MG703527	621b	Imbituba, SC	fresh	*Prionace glauca*	0.000	100	99
O22	MG703528	607b	Florianópolis, SC	fresh	*Prionace glauca*	0.000	97	100
FA02	MG703529	523b	Tramandaí, RS	fresh	*Prionace glauca*	0.002	100	99
FA03	MG703530	611b	Tramandaí, RS	fresh	*Prionace glauca*	0.000	96	100
FA23	MG703531	641b	Porto Alegre, RS	frozen	*Prionace glauca*	0.000	99	100
FA24	MG703532	588b	Porto Alegre, RS	frozen	*Prionace glauca*	0.002	100	99
FA25	MG703533	612b	Porto Alegre, RS	frozen	*Prionace glauca*	0.003	100	99
FA26	MG703534	634b	Porto Alegre, RS	frozen	*Prionace glauca*	0.000	100	100
FA27	MG703535	526b	Porto Alegre, RS	frozen	*Prionace glauca*	0.000	100	99
FA29	MG703536	556b	Porto Alegre, RS	frozen	*Prionace glauca*	0.000	100	100
FA31	MG703537	527b	Porto Alegre, RS	frozen	*Prionace glauca*	0.000	100	99
IIL26	MG703538	650b	Itajaí, SC	fresh	Rajiformes sp. BOLD AABB	0.000	96	100
E34^*^	MG703539	650b	Torres, RS	fresh	*Pseudobatos horkelii*	0.003	97	100
E36^*^	MG703540	610b	Torres, RS	fresh	*Pseudobatos horkelii*	0.002	98	100
E26^*^	MG703541	650b	Araranguá, SC	fresh	*Rhizoprionodon lalandii*	0.001	94	100
IIL13	MG703542	521b	Itajaí, SC	fresh	*Rhizoprionodon lalandii*	0.001	99	99
FA05	MG703543	590b	Imbé, RS	fresh	*Rhizoprionodon lalandii*	0.001	91	99
FA17	MG703544	512b	Porto Alegre, RS	fresh	*Rhizoprionodon lalandii*	0.001	100	99
O24	MG703545	650b	Florianópolis, SC	fresh	*Rhizoprionodon porosus*	0.001	95	100
E07^*^	MG703546	519b	Tramandaí, RS	fresh	*Sphyrna lewini*	0.024	100	100
E08^*^	MG703547	650b	Tramandaí, RS	fresh	*Sphyrna lewini*	0.021	98	99
E15^*^	MG703548	619b	Arroio do Sal, RS	fresh	*Sphyrna lewini*	0.022	97	100
E44^*^	MG703549	650b	Tramandaí, RS	fresh	*Sphyrna lewini*	0.021	97	99
MG04	MG703550	650b	Rio Grande, RS	fresh	*Sphyrna lewini*	0.034	97	99
MP55	MG703551	534b	Porto Alegre, RS	fresh	*Sphyrna lewini*	0.024	100	99
MP57	MG703552	542b	Porto Alegre, RS	fresh	*Sphyrna lewini*	0.023	97	100
MP58	MG703553	621b	Porto Alegre, RS	fresh	*Sphyrna lewini*	0.022	97	100
O06	MG703554	608b	Passo de Torres, SC	fresh	*Sphyrna lewini*	0.022	97	99
O07	MG703555	628b	Passo de Torres, SC	fresh	*Sphyrna lewini*	0.021	97	100
O08	MG703556	612b	Passo de Torres, SC	fresh	*Sphyrna lewini*	0.022	98	100
O09	MG703557	534b	Passo de Torres, SC	fresh	*Sphyrna lewini*	0.024	100	99
O27	MG703558	534b	Florianópolis, SC	fresh	*Sphyrna lewini*	0.024	100	100
O29	MG703559	650b	Florianópolis, SC	fresh	*Sphyrna lewini*	0.021	99	100
O28	MG703560	463b	Florianópolis, SC	fresh	*Sphyrna zygaena*	0.000	100	99
FA21	MG703561	642b	Porto Alegre, RS	fresh	*Shpyrna zygaena*	0.001	100	100
MP15	MG703562	630b	Porto Alegre, RS	fresh	*Squalus cubensis*	0.000	98	100
MP18	MG703563	603b	Porto Alegre, RS	fresh	*Squalus mitsukurii*	0.001	98	100
MP16	MG703564	641b	Porto Alegre, RS	fresh	*Squalus mitsukurii*	0.001	96	100
FA16	MG703565	593b	Porto Alegre, RS	fresh	*Squatina guggenhein*	0.001	96	100
MG08	MG703566	204b	Rio Grande, RS	fresh	*Squatina occulta*	0.000	95	100
IIL01	MG703567	650b	Itajaí, SC	frozen	*Xiphias gladius*	NC	100	99
IIL03	MG703568	650b	Itajaí, SC	frozen	*Xiphias gladius*	NC	100	99
IIL16	MG703569	650b	Itajaí, SC	frozen	*Xiphias gladius*	NC	100	99
IIL18	MG703570	589b	Itajaí, SC	frozen	*Xiphias gladius*	NC	100	99
IIL19	MG703571	458b	Itajaí, SC	frozen	*Xiphias gladius*	NC	100	100
IIL25	MG703572	622b	Itajaí, SC	frozen	*Xiphias gladius*	NC	100	100
E50^*^	MG703573	650b	Passo de Torres, SC	fresh	*Zapteryx brevirostris*	0.030	97	99
E54^*^	MG703574	232b	Passo de Torres, SC	fresh	*Zapteryx brevirostris*	0.000	100	100

a *Average genetic distance against all sequences from the same species in the final dataset*.

b *%Coverage and %Identity values considering the top-BLAST hit for the candidate species*.

* *All samples identified as E__ were obtained directly from fishing vessels, and were not purchased*.

*NC, not computed*.

### Laboratory procedures

DNA extraction started from a small portion (~100 mg) of the tissue. For most samples we used the Wizard® Genomic DNA Purification Kit (Promega) modified to include an initial digestion step with 200 μg proteinase k (Aljanabi and Martinez, 1997). For the remaining samples, we used a protocol based on the CTAB method (Doyle, 1987). We used the COI primers FishF2 (5′ TCG ACT AAT CAT AAA GAT ATC GGC AC 3′) and FishR2 (5′ ACT TCA GGG TGA CCG AAG AAT CAG AA 3′) (Ward et al., 2005). Amplification reactions were prepared with 0.4 μM of each dNTP, 1.5 mM MgCl_2_, 0.5 μM of each primer, 1 U Taq Polymerase, and ~40 ng of genomic DNA. Cycling conditions included an initial denaturing step of 94°C for 5′, followed by 10 cycles of 94°C for 1′, 55°C (−0.5°C/cycle) for 1′, and 72°C for 1′30″, and 30 additional cycles of 94°C for 1′, 50°C for 1′, and 72°C for 1′30″, with a final extension step of 72°C for 5′. The amplification products were visualized on a 1% agarose gel stained with GelRed™ (Biotium). PCR products were purified enzymatically using 0.33U SAP (Shrimp Alkaline Phosphatase) and 3.33U ExoI (Exonuclease I). PCR products were sequenced by the Sanger method in Macrogen Inc. (Seoul, South Korea) and Ludwig Biotec (Porto Alegre, Brazil). DNA sequencing was performed on both strands using the primers mentioned above.

### Data analysis

The consensus sequence for each sample was assembled and trimmed in Geneious 9.1 (www.geneious.com). The reliability of each consensus sequence was assessed by a thorough visual inspection of the chromatograms used in the assemblies to check for sequencing errors and artifacts. Low quality regions in the chromatograms, identified as a stretch of five or more contiguous bases having high background noise and uneven spacing, were trimmed and removed before sequence assembly. Because the assembly algorithm gives more weight to better quality reads, cases of sequence heterogeneity between strands are resolved in favor of the best quality read or, if both reads had similar quality for that position, marking it as an ambiguous base (N, R, Y, etc.). The consensus sequence was then used as a query for comparison with the NCBI database (http://www.ncbi.nlm.nih.gov/) using the Basic Local Alignment Search Tool—Nucleotide (BLASTn). In all cases, BLAST matched COI sequences from elasmobranchs (or, in some cases, from teleosts) with good coverage and identity (see Results), suggesting that we generated authentic COI sequences from our samples. We recorded the species representing the top BLAST hit for each query. Following this, we built a dataset of 2,877 COI sequences deposited in the GenBank including all species of all genera represented in the list of top BLAST hits. For example, if the top BLAST hit for a given sample was *S. lewini*, we included all sequences from all *Sphyrna* species (including eventual “*Sphyrna* sp.” entries) in the dataset. We then picked at random 2–8 sequences for each species, which were aligned with the consensus sequences from the samples generated in this study using MAFFT 7.0 (Katoh and Standley, 2013), leading to a final dataset of 323 COI sequences for 147 species (including undescribed or unknown species). As a final quality control step, we checked the dataset for nonsense mutations and alignment gaps, as both could indicate the presence of nuclear mitochondrial translocations (Numts) (Triant and DeWoody, 2007). The final alignment file can be downloaded as Supplementary Material (File S1). The best substitution model (HKY+G+I) for this final dataset was estimated in jModelTest 2 based on the corrected Akaike Information Criterion (AICc) (Darriba et al., 2012). Pairwise genetic distances was estimated in PAUP^*^ 4.0 (Swofford, 2002) based on most likely substitution model and its associated parameters [Lset base = (0.3624 0.2434 0.0914) nst = 2 tratio = 6.1561 rates = gamma shape = 0.8490 ncat = 4 pinvar = 0.4860]. For this final dataset, we inferred the maximum likelihood (ML) tree in RAxML 8.2.10 (Stamatakis, 2014). Node credibility was assessed based on 1,000 bootstrap replicates.

## Results

In total, 63 samples were collected, amplified, sequenced, and compared to GenBank sequences (Table 1). High quality sequences ranged between 204 and 650 bases. There was no sequence heterogeneity between strands involving high quality bases from two or more reads. Overall, our analysis suggests the presence of 20 different species among the samples. Seven samples were identified as belonging to two Actinopterigii (ray-finned fishes) species: *Xiphias gladius* (Perciformes, swordfish; *n* = 6), and *Genidens barbus* (Siluriformes, white sea catfish; *n* = 1). The remaining samples may represent 18 elasmobranch species from three shark orders (Carcharhiniformes, Squaliformes, and Squatiniformes; *n* = 42, 3, 2, respectively) and four ray orders (Rhinopristiformes, Myliobatiformes, Rajiformes, and Torpediniformes; *n* = 4, 3, 1, 1, respectively). Three ray species (*P. horkelii, Z. brevirostris*, and *N. brasiliensis*) were only found in samples from fishing vessels (i.e., they were not purchased in the market).

Based on COI sequences, all but one elasmobranch samples were identified at the species level, representing 17 formally described species. One sample was associated with an undescribed or unsequenced species (which occurs in GenBank as “Rajiformes sp. BOLD: AABB1882”). The most common species found among market samples were *Prionace glauca* (blue shark, *n* = 15) and *S. lewini* (scalloped hammerhead shark, *n* = 14). All other species were far less common, including *R. lalandii* (Brazilian sharpnose shark, *n* = 4), *Carcharhinus brachyurus* (copper shark, *n* = 3), *Carcharhinus falciformis* (silky shark, *n* = 2), *Sphyrna zygaena* (smooth hammerhead shark, *n* = 2), *Squalus mitsukurii* (shortspine spurdog, *n* = 2), *Galeorhinus galeus* (school shark, *n* = 1), *Rhizoprionodon porosus* (Caribbean sharpnose shark, *n* = 1), *Squalus cubensis* (Cuban dogfish, *n* = 1), *Squatina occulta* (hidden angel shark, *n* = 1), and *Squatina guggenheim* (spiny angel shark, *n* = 1). All ray species identified in the study occurred once or twice among the samples: *G. altavela* (spiny butterfly ray, *n* = 2), *P. horkelii* (Brazilian guitarfish, *n* = 2), *Z. brevirostris* (shortnose guitarfish, *n* = 2), *Myliobatis goodei* (southern eagle ray, *n* = 1), and *N. brasiliensis* (Brazilian electric ray, *n* = 1).

**Figure 2:**
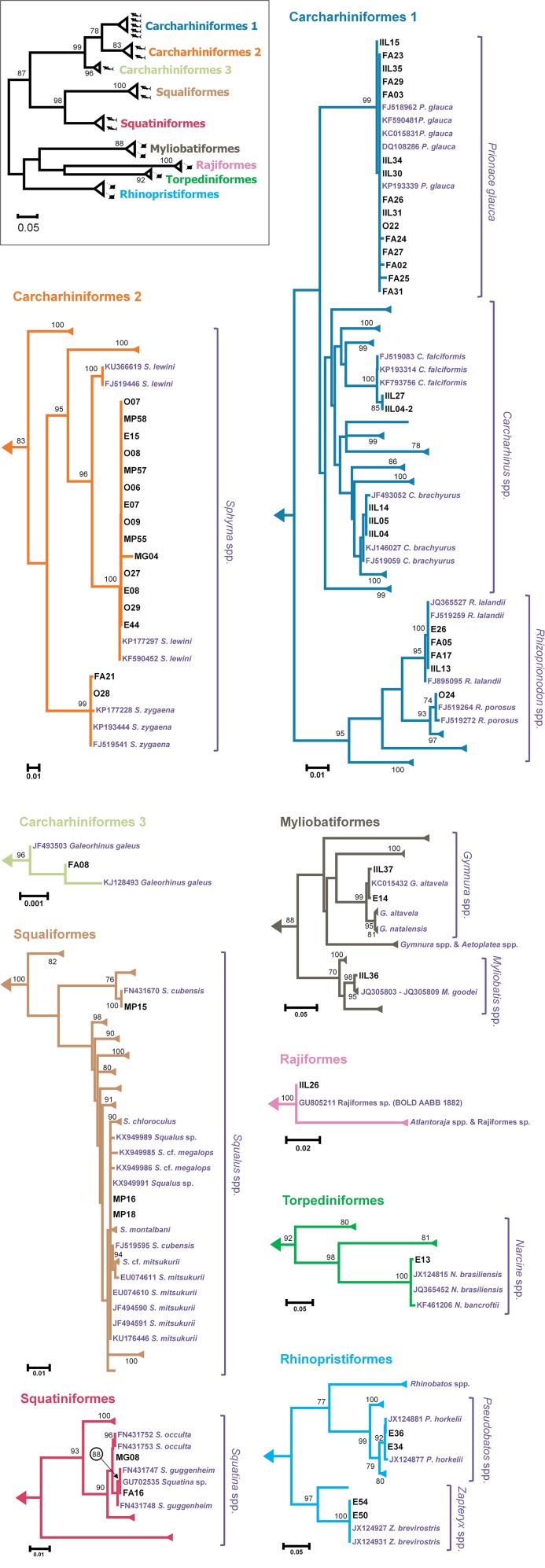
ML tree based on HKY+G+I distance. The miniature on the upper left side shows major groups, displayed in more detail in individual panels. The number of shark and ray symbols represent the number of different species identified in the study for each group. Please note that this is an unrooted tree. Most entries were collapsed and the names were omitted for clarity. Samples from the present study are labeled according to Table 1. The most likely candidate species, together with other closely related species are shown in red. The numbers above the branches represent bootstrap percentage based on 1,000 replicates. Bootstrap values <70 were omitted. Please note the different scale among panels. The full ML tree is available as Supplementary Material (File S3).

The average genetic distance between each sample and representatives of its most likely candidate species (determined by its clustering in the ML tree) was always lower than 3.50%, and usually lower than 1% (Table 1). The ML tree showed cohesive clusters of conspecific sequences (Figure 2). The few exceptions, which had bootstrap support values lower than 90, included *S. mitsukurii, C. brachyurus, S. guggenheim*, and *S. occulta* (Figure 2). In all cases, however, the estimated genetic distance between our samples and reference sequences were used to indicate the most likely candidate species (shown in Table 1). An interesting case is sample MP16, whose top-hit in BLAST was *Squalus montalbani*, but clustered with *S. mitsukurii* in the ML tree (Figure 2). However, both MP16 and MP18 showed a much smaller distance from *S. mitsukurii* (0.0009) than to any other closely related species (0.0027 vs. *S. cf. megalops*; 0.0043 vs. *S. montalbani*; 0.0058 vs. *S. chloroculus*; and 0.0088 vs. *S. cf. mitsukurii*). Similarly, IIL04, IIL05, and IIL14 were much closer to *C. brachyurus* (0.0014) than to *C. brevipinna* (0.0150), MG08 was closer to *S. occulta* (0.0000) than to *S. guggenheim* (0.0071), while FA16 was closer to *S. guggenheim* (0.0008) than to *S. occulta* (0.0064). The complete distance matrix can be downloaded as Supplementary Material (File S2).

## Discussion

We found 18 Elasmobranchii and two Actinopterigii species among the samples acquired in Southern Brazilian fish markets as “cação,” “caçonete,” or “filé anjo.” This represents 17% of all elasmobranch species registered for Southern Brazil and 13% of the species described for Brazil (Bornatowski et al., 2009). Other studies, based on other molecular markers, that aimed at species identification of shark filets from Northern Brazil have also shown the great number of species being trade without any taxonomic control (Rodrigues-Filho et al., 2009; Palmeira et al., 2013). Unfortunately, our DNA data does not allow us to conclude that these samples represent individuals captured in Southern Brazil. For example, most individuals included in the final dataset did not have location information. Additionally, even if this was available, it is unclear whether COI would have enough resolution to allow unambiguous recognition of regional stocks for these species (Antoniou and Magoulas, 2014). However, the fact that the vast majority of samples collected in this study were purchased fresh is a strong indication that these specimens may have been captured off Southern Brazil or in nearby areas.

The use of the COI DNA barcode allowed us to identify all samples at the specific level even though some cases deserve further discussion. The best match for IIL26 was an undescribed or unsequenced species, Rajiformes sp. BOLD:AAB1882 (Coverage = 96%, Identity = 100%). The sample MG08 resulted in a short DNA sequence, whose top-result in BLAST was against *S. occulta* (Coverage = 95%, Identity = 100%), but showed an inconclusive clustering with any *Squatina* species in the ML tree (Figure 2). Nevertheless, as occurred for other samples (FA16, IIL04, IIL05, IIL14, MP16, MP18), comparing the genetic distance among alternative candidate species allowed the identification of the most likely candidate for each sample. In the case of the samples associated to *Squatina*, species identification was corroborated by the fact that both *S. occulta* and *S. guggenheim* occur off Southern Brazil (Vaz and Vaz and De Carvalho, 2013) and that both samples were acquired as fresh filets, likely indicating a local catch. With a single exception, the species associated with the top-BLAST result also resulted in the lowest average genetic distance. The exception was MG16, whose top-BLAST result was *Squalus montalbani* (Coverage = 100%, Identity = 99%), but whose lowest average genetic distance was against *S. mitsukurii*, which also represented the second and third top-BLAST results (Coverage = 96%, Identity = 100%). The low genetic distance among *Squalus* species and the lack of a clear structure in the ML tree (Figure 2) may indicate that DNA barcoding for this genus may be more complicated than for other genera, and may require other genetic markers. From the taxonomic point of view, it is difficult to discriminate among *Squalus* species (Haddad and Gadig, 2005), which may be due to a shallow diversification time that is reflected in the low genetic distances among several species. The inherently difficult taxonomy of the genus may favor misnomers in reference databases. In this regard, *S. cubensis* presents a likely example of database confusion. There are two COI sequences for this species in GenBank. However, while the entry FJ519595 is close to *S. mitsukurii* (~0.2% genetic distance) the other, FN431670, is distantly related to it (~7.2% genetic distance) and associated with sample MP15 (Figure 2). These issues reinforce the importance of database curation and maintenance, with rigorous taxonomic criteria for the deposition of reference sequences (Ekrem et al., 2007; Teletchea, 2009; Dudgeon et al., 2012). It also highlights that in some cases it may be important to analyze additional genetic markers for a more accurate species identification (Mendonça et al., 2009; Moftah et al., 2011; Pérez-Jiménez et al., 2013).

The most abundant shark species in our samples were *Prionace glauca* and *S. lewini* (23.8 and 22.2%, respectively). *P. glauca* is distributed globally and its capture volume has been estimated at approximately 20 million individuals per year (Mendonça et al., 2012). Despite its endangered status (Table 2), *P. glauca* is the most fished shark in the world, representing 56% of the total catch of pelagic sharks, especially by industrial fisheries in which the target species are tuna or swordfish (Rose, 1996; Dulvy et al., 2003; Camhi et al., 2009). After the increasing market demand for shark fins and the high prices paid for them, these animals began to be targeted for the removal of these parts, with the carcasses being sold worldwide (Domingues, 2011). Indeed, we identified *P. glauca* in all samples acquired as frozen filets, which may reflect that these individuals were captured in other parts of the world, such as Taiwan and subsequently imported to Brazil (Figure S1). However, we also found *P. glauca* among fresh samples, which more likely indicates local capture. On the other hand, *S. lewini* was the most abundant species among fresh samples, which may indicate a higher local impact on this species. Several authors have raised concerns of predatory fishing for this species off Brazil due to the high commercial value of its fins (Amorim et al., 2011). This results in fishing pressures occurring over all phases and life cycles of these animals, including neonates (Mader et al., 2007) both on the continental shelf and in oceanic waters (Kotas, 2004; Kotas et al., 2005; Vooren and Klippel, 2005).

**Table 2:** Conservation status (global, national, and regional) of the species found in this study.

**Species**	**Common name^a^**	**IUCN^b^**	**ICMBio^c^**	**RS^d^**	**SC^e^**
*Carcharhinus brachyurus*	cooper shark	NT 2003	DD^*^	–	–
*Carcharhinus falciformis*	silky shark	NT 2016	NT^*^	–	–
*Galeorhinus galeus*	school shark	VU 2006	CR	CR	–
*Gymnura altavela*	butterfly ray	VU 2007	CR	EN	–
*Myliobatis goodei*	southern eagle ray	DD 2009	CR	CR	–
*Narcine brasiliensis*	Brazilian electric ray	DD 2007	DD^*^	–	–
*Prionace glauca*	blue shark	NT 2009	NT^*^	VU	–
*Pseudobatos horkelli*	Brazilian guitarfish	CR 2016	CR	CR	CR
*Rhizoprionodon lalandii*	Brazilian sharpnose shark	DD 2004	NT^*^	–	–
*Rhizoprionodon porosus*	Caribbean sharpnose shark	LC 2006	DD^*^	–	–
*Sphyrna lewini*	scalloped hammerhead shark	EN 2007	CR	CR	EN
*Sphyrna zygaena*	smooth hammerhead	VU 2005	CR	CR	EN
*Squalus cubensis*	Cuban dogfish	DD 2006	–	–	–
*Squalus mitsukurii*	shortspine spurdog	DD 2007	DD^*^	–	–
*Squatina guggenheim*	spiny angel shark	EN 2007	CR	CR	EN
*Squatina occulta*	smoothback angel shark	EN 2007	CR	CR	–
*Zapteryx brevirostris*	shortnose guitarfish	VU 2006	VU	CR	–

* *Species included in the National List of Species Threatened of Extinction (available at Portaria MMA n° 445 of 2014)*.

a *IUCN Red List of Threatened Species (IUCN, 2017)*.

b *Global conservation status according to IUCN (2017) criteria, followed by the year of assessment: (CR) critically endangered; (EN) endangered; (VU) vulnerable; (NT) near threatened; (DD) data deficient; (NE) not evaluated*.

c *National conservation status according to the Brazilian Red Book of Threatened Faunal Species (Instituto Chico Mendes de Preservação da Biodiversidade, 2016)*.

d *Regional conservation status according to the List of Threatened Fauna of the Rio Grande do Sul State (Fundação Zoobotânica e Secretaria do Ambiente Desenvolvimento Sustentável, Decreto n° 51.797)*.

e *Regional conservation status according to the List of Threatened Fauna of the Santa Catarina State (Fundação de Meio Ambiente – FATMA)*.

Regarding their conservation status, IUCN estimates that 47% of the elasmobranch species found in this study are considered threatened at the global level, 53% are threatened at the national level, and 47% are critically endangered at the national level (Table 2). It is difficult, however, to present a more regional picture, given that the red list for both Rio Grande do Sul and Santa Catarina states include only 59 and 23.5% of the species identified in this study, even though there are records for most of these species off these Brazilian states (Gadig, 2001). The conservation status for *R. lalandii, S. mitsukurii, S. cubensis, M. goodei*, and *N. brasiliensis* is unknown due to data deficiency (DD). In the worst-case scenario, ~50% of the species identified in this study would be threatened to some extent.

Our sampling was restricted to the south of Brazil due to a limited budget, but it would be important to perform similar studies in other Brazilian regions to provide a better picture of the shark fishing and trade in the country. It should be noted, however, that the Southern coast of Brazil is a hotspot for shark diversity, with high species richness, high endemism, and functional richness (Lucifora et al., 2011). Another future direction would be investigating how much of the shark meat market involves individuals fished locally.

Finally, an important issue in the conservation of these species is how local human populations will engage in more sustainable consumption practices. In this sense, labeling the meat of any shark species as “cação” may impose major barriers to conservation measures for this group, allowing the inadvertent consumption of protected species (Jacquet and Pauly, 2008). Indeed, Bornatowski et al. (2015), who interviewed fish meat consumers in Southern Brazil, reported that 61% of respondents claimed that they have never tried shark meat, even though they ate “cação.” In addition, 69% of respondents said they did not know that at least 25% of all elasmobranchs are threatened. Given these answers, it is evident that a significant portion of the population buying these products is not aware of the impact of their consumption habits, or of the current conservation status of elasmobranch species. Another issue for consumers is mislabeling of shark products, a common outcome of DNA barcode assessments of seafood products (Barbuto et al., 2010; Filonzi et al., 2010). This is illustrated by the presence of the two teleost species detected among our sample (Table 1). Therefore, it becomes essential and an ethical responsibility for the industry to label their products correctly and allow informed decision-making by the consumers. We suggest that all meat being sold as “cação” should be accompanied by the species common name, followed by its scientific name, and, whenever possible, the species threat categories according to the IUCN Red List. While fishing legislation may also have a positive impact on natural populations by suspending the capture and marketing of endangered elasmobranchs, environmental education measures focusing on the fishing community and on consumers will be fundamental for the effective protection of these species.

## Author contributions

FA-S, CS, LO, VV, and NF designed the study; FA-S, CS, CC, PF, and RM executed experimental procedures; FA-S, CS, PF, LG, LO, VV, and NF performed data analysis and interpretation; FA-S, CS, VV, and NF wrote the paper.

### Conflict of interest statement

The authors declare that the research was conducted in the absence of any commercial or financial relationships that could be construed as a potential conflict of interest.
