# Supplementary material for: Molecular Identification of Shark Meat From Local Markets in Southern Brazil Based on DNA Barcoding: Evidence for Mislabeling and Trade of Endangered Species
Source: Front Genet. 2018 Apr 27;9:138. doi: 10.3389/fgene.2018.00138 (PMC5934587; doi:10.3389/fgene.2018.00138)
Supplement: Supplementary file 1 [file Image_1.PDF]

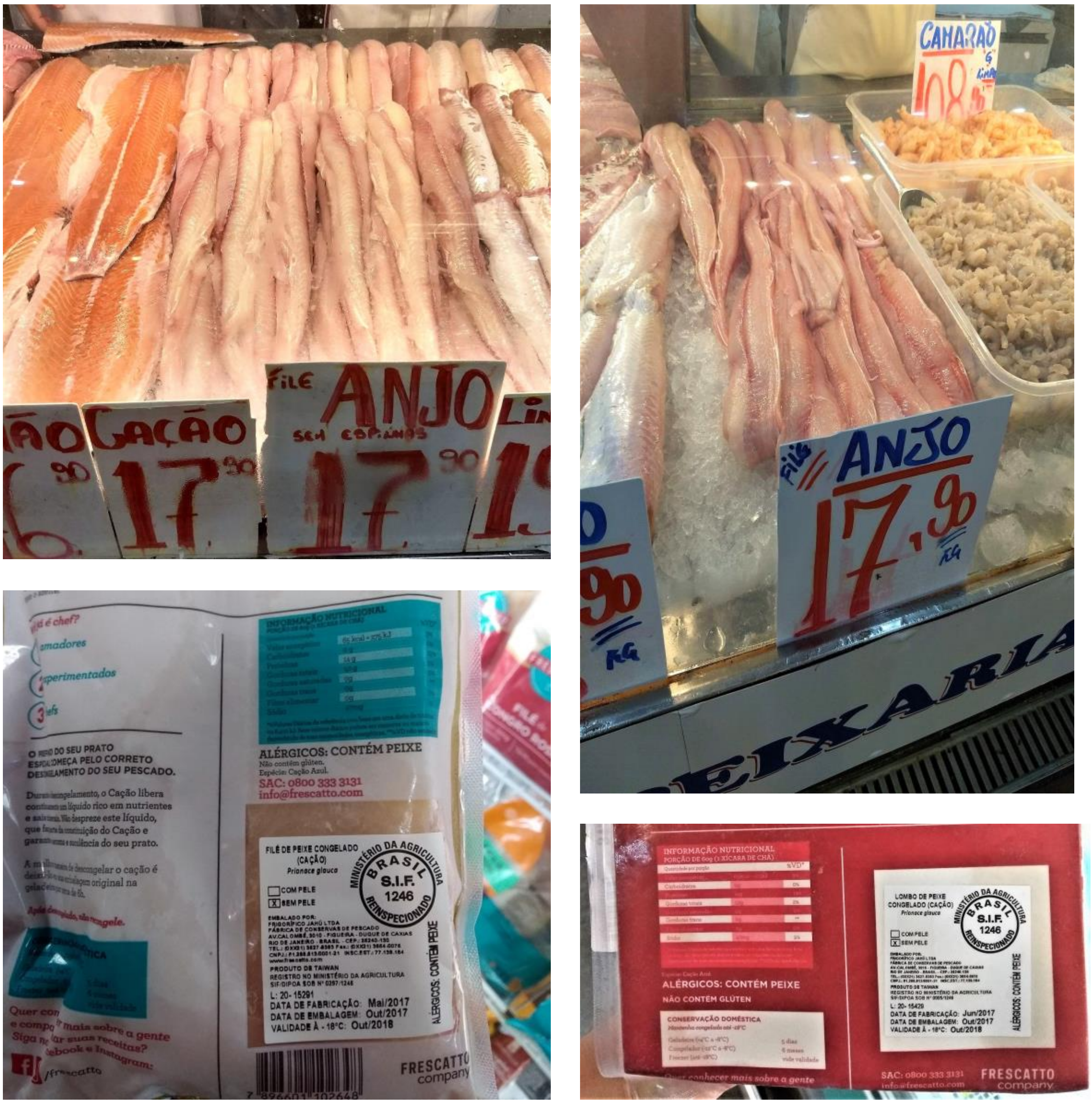

Figure S1 – Examples of shark meat labelled as "cação" or "anjo" from fish markets and in supermarkets in Southern Brazil. Note that in some cases, a species name may appear on the label, especially for frozen fillets.
